# Making plaque assessment easier – a validation study of simplified versions of the Marginal Plaque Index

**DOI:** 10.1186/s12903-024-05168-8

**Published:** 2024-11-14

**Authors:** Ulrike Weik, Zdenka Eidenhardt, Renate Deinzer

**Affiliations:** Department of Medicine, Justus-Liebig-University Giessen, Klinikstr. 29, 35392 Giessen, Germany

**Keywords:** Dental Plaque Index, Oral Hygiene Index, Oral Hygiene, Dental plaque, Toothbrushing, Validation Studies as Topic

## Abstract

**Background:**

The assessment of plaque indices may be time-consuming and error-prone. Simplification of these indices may increase their utility without compromising their validity. The aim of this study was to evaluate the validity of two simplified versions of the Marginal Plaque Index (MPI).

**Methods:**

Two simplified versions of the MPI as well as the Plaque Control Record (PCR) were derived from full-scale MPI assessments in two studies with four age groups (*N* = 42 10-year-olds; *N* = 24 15 year-olds; *N* = 53 university students (18y-33y); *N* = 66 parents (32y-57y). Correlations with the Turesky modification of the Quigley-Hein Index (TQHI) and the Papillary Bleeding Index (PBI) were calculated.

**Results:**

The indices derived from the MPI showed high convergence with each other (all r ≥ 0.94) and with the TQHI (r ≥ 0.80). The concurrent validity of the MPI with the PBI was equal to that of the TQHI in all age groups. The simplified versions of the MPI and the PCR show a lower convergent validity with the PBI than the MPI within parents (*p* < 0.05). In the other age groups, their convergent validity was equal to that of the MPI.

**Discussion:**

Simplification of the MPI does not affect its convergent validity with other plaque indices but may reduce its concurrent validity with the PBI in middle-aged adults.

## Background

The evaluation of plaque is of significant importance in the assessment of oral health, the identification of oral disease risk, and the promotion of effective oral hygiene practices. A number of plaque indices exist that assess the extent of plaque accumulation.

Some indices rely on the assessment of plaque over the entire tooth including the Refinement of the Modified Navy Plaque Index (RMNPI; [1]) and the Turesky modification of the Quigley and Hein index (TQHI; [2, 3]). The RMNPI divides each surface of a tooth into nine segments and assesses the presence or absence of plaque in these segments. This approach is time-consuming and lacks clarity regarding the precise boundaries of each segment, which can lead to measurement inaccuracies. The TQHI is a widely used index that scores on both lateral surfaces of each tooth how far plaque extends to the crown. Plaque confined to the gingiva is scored 1 or 2, while scores 3–5 refer to plaque that covers one, two, or three-thirds of the crown. This assessment requires extensive training in order to achieve good interrater reliability. The mean value of the sores of all teeth and surfaces forms the TQHI as an expression of the overall oral hygiene. This index thus weighs plaque at the upper parts of the crown higher than plaque at the gingival margin.

**Fig. 1:**
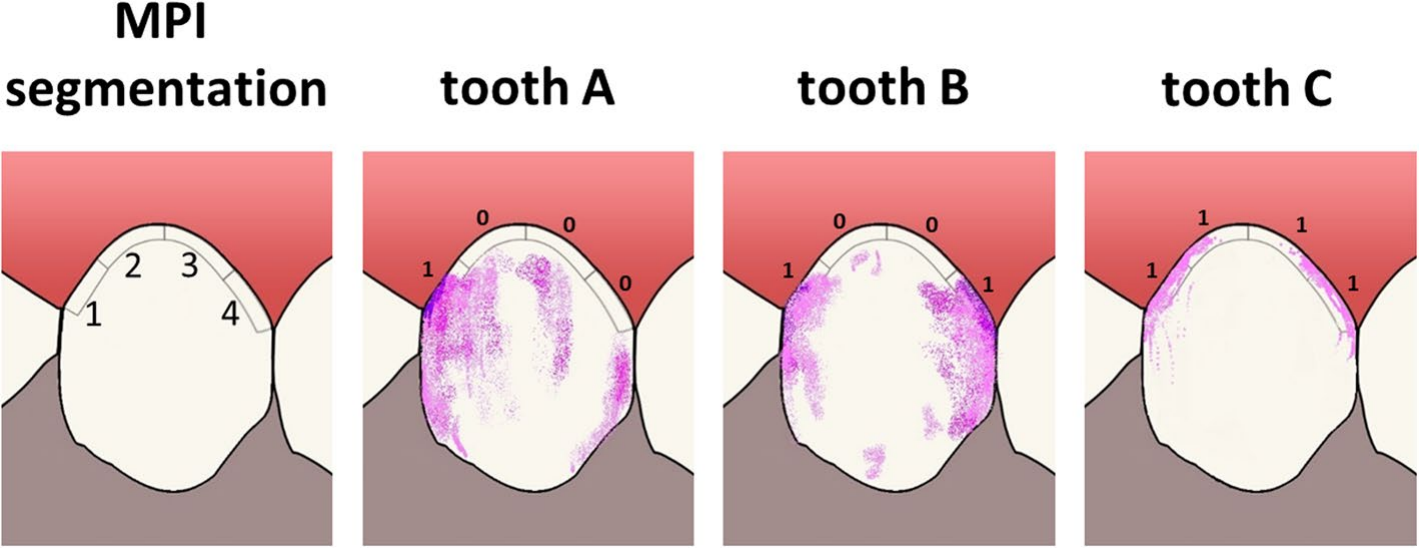
Marginal Plaque Index (MPI, 5). Segmentation of the gingival margin into four equal segments (1 = distal, 2 = cervico-distal, 3 = cervico-mesial, 4 = mesial). Teeth **A-C** show stained plaque: **A** = segment 1 is scored as 1 (= plaque) as there is visible plaque, segments 2–4 are scored as 0 (= no plaque); **B** = segments 1 and 4 are scored as 1, segments 2 and 3 are each scored as 0; **C** = all four segments at the gingival margins show plaque and are each scored as 1

Other indices assess plaque only at the gingival margin, including the Plaque Control Record (PCR; [4]) and the Marginal Plaque Index (MPI; [5]). These indices are of particular interest when dealing with periodontal diseases such as gingivitis and periodontitis, as these inflammatory conditions emerge at the gingival margin [6–8]. The MPI and PCR are also straightforward methods for assessing plaque, as they simply identify the presence (score 1) or absence (score 0) of plaque in clearly defined segments of the dentogingival junction. The percentage of segments scoring 1 is indicative of the extent of plaque at the gingival margin. The PCR assesses plaque in four segments per tooth: proximal mesial, proximal distal, oral and vestibular (O`Leary et al., 1972). However, in order to ascertain the presence or absence of plaque at the proximal area, it is necessary to evaluate both the buccal and palatinal surfaces simultaneously. This process is time-consuming and laborious, requiring the examiner to frequently alter the viewing angle and lighting conditions. The MPI is more convenient in this respect and simultaneously permits a more differentiated assessment of plaque distribution. The method divides the area adjacent to the gingival margin of each tooth surface into four segments of equal size: distal, cervico-distal, cervico-mesial and mesial. This results in eight clearly defined segments per tooth (for further details refer to to Fig. 1). The index can be expressed as an overall measure incorporating all segments, or alternatively, as a measure specifically evaluating oral hygiene at the proximal (distal/mesial) and cervical segments, respectively. Validation studies have demonstrated that index’s capacity to discern changes in interdental hygiene and brushing technique [5]. However, the process of evaluating plaque in eight segments per tooth is time-consuming and produces a significant amout if data. The question thus arises as to whether it is possible to reduce the number of segments assessed while maintaining the validity of the index. However, reducing the information in a measure reduces its reliability. This could then affect its validity. The present study therefore aims to evaluate the validity of two simplified versions of the original MPI by comparing them with the original MPI, the PCR and the TQHI. It tests the following hypotheses: (1) that the simplified versions of the MPI have a high convergent validity with the MPI, the PCR, and the TQHI; and (2) that the concurrent validity of the MPI with the Papillary Bleeding Index (PBI; [9]) differs from that of the other indices with the PBI.

## Materials and methods

The current study uses data from previously published studies that measured plaque by the use of the MPI [5]. It calculates the values of simpler indices based on these measurements.

### Participants and procedures

This article uses data collected from four different age groups within two studies. Detailed information on the studies has already been provided elsewhere [10–13]. In brief, the trials conformed to the Declaration of Helsinki and ethical permissions were ethically approved by the Ethics Committee of the Medical Faculty of the Justus Liebig University of Giessen, Germany (No: 255/18; No: 254/18). They were conducted under highly standardised conditions by dentists in the dental examination rooms of the Institute of Medical Psychology at the Justus Liebig University Giessen. The participants of the first study (study duration April 2019 to July 2019) were 53 university students, aged 18–33 years [10]. The participants of the second study (study duration August 2019 to December 2019) were 42 ten-year-olds and 24 fifteen-year-olds, each of whom was examined with one parent whose age ranged from 33 to 57 years [11–13]. Study participants were asked to brush their teeth "to the best of their ability" and dental plaque was assessed after brushing. Table 1 shows demographics, dental (decayed, missing and filled teeth; DMFT/dmft) and gingival status (PBI), and plaque levels (MPI; TQHI) after thorough toothbrushing for the four age groups. All participants were habitual manual toothbrush users; they had no cognitive or physical impairments that might affect toothbrushing, and did not wear fixed orthodontic appliances, removable dentures/prostheses, or piercings/tooth jewellery, and had no professional tooth cleaning within the last 4 months.

**Table 1:** Characteristics of the four age groups

	**10-year-olds**^**a**^ (*n* = 42)	**15-year-olds**^**a**^ (*n* = 24)	**Students**^**b**^ (*n* = 53)	**Parents**^**a**^ (*n* = 66)
	M ± SD n/n			
**Demographic data**				
female/male/non-binary	21/21/ -	6/17/1	43/9/1	54/11/1
age	10.14 ± 0.52	15.17 ± 0.38	22.87 ± 2.56	44.47 ± 5.34
**Dental status**^**c**^				
dmf-t, DMF-T	1.50 ± 2.47	2.25 ± 2.63	2.72 ± 3.39	13.05 ± 5.15
**Gingival status**^**d**^				
PBI mean	0.83 ± 0.43	0.85 ± 0.49	0.71 ± 0.39	0.97 ± 0.51
PBI % bleeding full mouth	50.46 ± 21.54	49.86 ± 23.43	42.64 ± 18.97	51.50 ± 19.04
**Plaque after brushing**^**d**^				
MPI	81.86 ± 14.36	71.64 ± 17.15	62.34 ± 15.63	69.71 ± 15.25
TQHI mean	2.83 ± 0.59	2.44 ± 0.75	2.05 ± 0.48	2.40 ± 0.56

^a^[11–13]; ^b^[10] ^c^Without 3rd molars. ^d^Including 3rd molars

### Data collection: clinical measures

All clinical data were assessed by trained and calibrated dentists on all existing teeth (including 3rd molars). Calibration was considered successful if more than 90% of the scores agreed and less than 10% of the scores differed by more than one score unit in five consecutive training participants who were not included in the studies. Prior to plaque assessment dentists assessed the PBI ([9], modified by [14]) on the outer (buccal/labial) and inner (palatinal/lingual) surfaces, which serves in the present analysis as an indicator of gingivitis. The PBI scores range from 0 to 4 (0 = no bleeding on probing; 1 = single bleeding spot; 2 = multiple bleeding spots or thin band; 3 = interdental triangle filled with blood; 4 = heavy bleeding on probing).

**Fig. 2:**
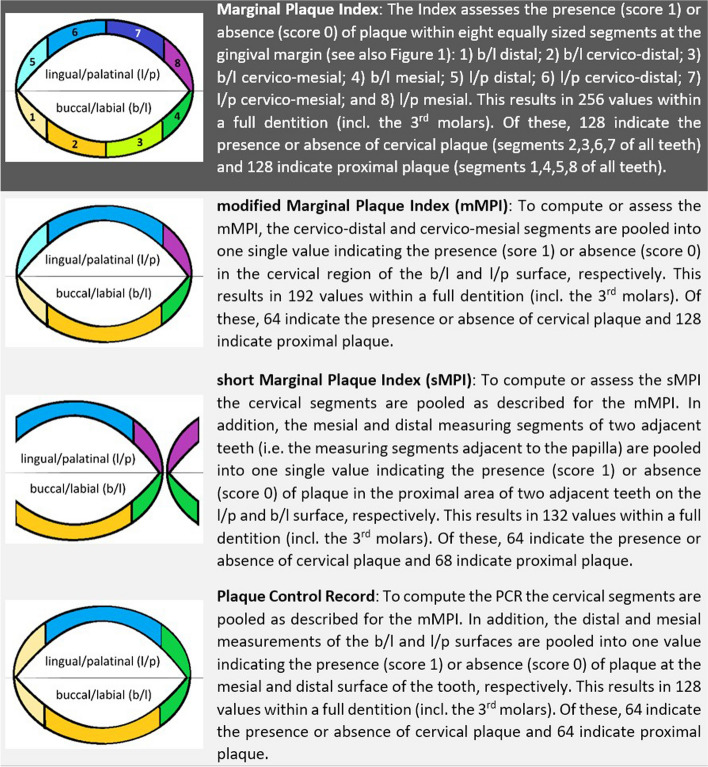
Plaque indices analyzed. The Figure shows schematically the gingival margin of one (MPI, mMPI, PCR) or two adjacent (sMPI) teeth and how the mMPI, the sMPI and the PCR are derived from the MPI data. For each differently coloured segment, the presence (score = 1) or absence (score = 0) of plaque is assessed. The index for the whole dentition is then calculated as the percentage of segments scoring 1

For plaque assessment, the dentists dried the teeth with an air stream and then applied a plaque-disclosing solution (Mira-2-Ton®; Hager & Werken, Duisburg, Germany). They assessed the MPI [5] described in detail in Fig. 1 and 2, and the TQHI. The TQHI assesses plaque on the entire dental crown both at outer (buccal/labial) and inner (lingual/palatinal) surfaces. The following scores are applied ([2], p. 41):

- 0 = No plaque;
- 1 = Separate flecks of plaque at the cervical margin of the tooth;
- 2 = A thin continuous band of plaque (up to one mm) at the cervical margin of the tooth;
- 3 = A band of plaque wider than one mm but covering less than one-third of the crown of the tooth; 4 = Plaque covering at least one-third but less than two thirds of the crown of the tooth;
- 5 = Plaque covering two-thirds or more of the crown of the tooth.

### Computation of indices from the MPI values

The raw MPI data, which indicate the presence or absence of plaque for eight segments per tooth, were used to calculate two simplified versions of the MPI and the PCR [4] as an additional reference. Figure 2 illustrates how the different measures were derived from the MPI data and how they can be assessed in the future. It also shows how many individual plaque values need to be recorded in order to generate each index.

### Statistical analysis

Statistical analyses were performed using a statistical software package (IBM Corp. Released 2022. IBM SPSS Statistics for Windows, Version 29.0. Armonk, NY: IBM Corp).

Correlations were calculated to determine the convergence between the different plaque indices, including the TQHI as an additional criterion. The criterion validity of the plaque indices regarding gingivitis was also assessed by examining how they correlated with the PBI. Correlations were calculated as Pearson and Spearman correlations. Spearman correlations refer to rank-scaled values and thus control for artefacts caused by outlying values or violations of the normal distribution assumption. To compare the correlations the MPI has with the PBI with those of the other measures with the PBI the formulas provided by Eid et al. for comparing correlations within groups were applied [15]. This was done only for Pearson correlations and only in cases where either the visual inspection of the distribution of the data or the Kolmogorov–Smirnov goodness of fit test (*p* = 0.05) indicated no deviation from the normal distribution of the variables involved.

## Results

### Descriptive statistics

Table 2 shows descriptive data of the original MPI, the simplified versions mMPI and sMPI, the PCR index derived from the MPI data and the TQHI in the groups of 10-year-olds, 15-year-olds, students and parents, respectively. All indices refer to the entire dentition.

**Table 2:** Descriptive statistics of the plaque indices in the four groups

	**10-year-olds (*n* = 42)**		**15-year-olds (*n* = 24)**		**University students (*n* = 53)**		**Parents (*n* = 66)**	
	Mean	SD	Mean	SD	Mean	SD	Mean	SD
**MPI**	**81.86**	14.36	**71.64**	17.15	**62.34**	15.63	**69.71**	15.25
**mMPI**	**86.93**	12.35	**78.72**	16.33	**70.34**	14.80	**77.01**	15.12
**sMPI**	**87.63**	11.59	**79.91**	15.90	**71.25**	13.68	**77.16**	14.38
**PCR**	**88.76**	11.14	**82.81**	13.13	**73.48**	13.24	**77.91**	14.34
**TQHI**	**2.83**	0.59	**2.44**	0.75	**2.05**	0.48	**2.40**	0.56
**PBI**	**0.83**	0.43	**0.85**	0.49	**0.71**	0.39	**0.97**	0.50

Tables 3, 4, 5, 6 present the results of the correlation analyses. Comparisons of the Pearson and the Spearman correlations do not reveal any artefacts caused by outlying values.

**Table 3:** Inter-correlations of the MPI indices and TQHI in 10 and 15-year-olds

	**MPI**		**mMPI**		**sMPI**		**PCR**		**TQHI**	
	r	rho	r	rho	r	rho	r	rho	r	rho
	***10-year-olds (n* = *42)***									
**MPI**	**–**	**–**	**0.98**	0.98	**0.97**	0.95	**0.95**	0.92	**0.82**	0.82
**mMPI**	**0.98**	0.98	**–**	**–**	**0.97**	0.95	**0.96**	0.92	**0.81**	0.83
**sMPI**	**0.97**	0.96	**0.96**	0.95	**–**	**–**	**0.99**	0.98	**0.77**	0.79
**PCR**	**0.96**	0.92	**0.94**	0.90	**0.99**	0.95	**–**	**–**	**0.76**	0.76
**TQHI**	**0.92**	0.90	**0.94**	0.93	**0.88**	0.84	**0.85**	0.80	**–**	**–**
	***15-year-olds (n* = *24)***

**Table 4:** Inter-correlations of the MPI indices and TQHI in Students and Parents

	**MPI**		**mMPI**		**sMPI**		**PCR**		**TQHI**	
	r	rho	r	rho	r	rho	r	rho	r	rho
	**University students (*n* = 53)**									
**MPI**	**–**	**–**	**0.99**	0.99	**0.98**	0.98	**0.97**	0.97	**0.90**	0.88
**mMPI**	**0.98**	0.97	**–**	**–**	**0.98**	0.97	**0.96**	0.94	**0.90**	0.89
**sMPI**	**0.98**	0.97	**0.98**	0.96	**–**	**–**	**0.98**	0.97	**0.86**	0.84
**PCR**	**0.96**	0.96	**0.95**	0.93	**0.98**	0.98	**–**	**–**	**0.84**	0.80
**TQHI**	**0.87**	0.83	**0.87**	0.84	**0.84**	0.79	**0.82**	0.78	**–**	**–**
	**Parents (*n* = 66)**

**Table 5:** Correlations between PBI and different plaque indices

	**10-year-olds (*n* = 42)**		**15-year-olds (*n* = 24)**		**University students (*n* = 53)**		**Parents (*n* = 66)**	
	r	rho	r	rho	r	rho	r	rho
**MPI indices**								
MPI	**0.49**	0.51	**0.63**	0.61	**0.52**	0.52	**0.45**	0.35
mMPI	**(0.50)**	0.53	**(0.59)**	0.62	**0.53**	0.53	**0.38**^**a**^	0.34
sMPI	**0.45**	0.46	**(0.58)**	0.58	**0.49**	0.46	**0.38**^**a**^	0.30
**PCR**	**0.45**	0.43	**0.56**	0.55	**0.49**	0.47	**0.35**^**a**^	0.28
**TQHI mean**	**0.57**	0.60	**0.73**	0.69	**0.54**	0.50	**0.46**	0.31

^a^significantly different from MPI; Brackets indicate that the assumption of normal distribution is violated and that the respective correlations are not compared with those of the MPI with the PBI

**Table 6:** Correlations between PBI and subscales of the MPI indices

	**10-year- olds (*n* = 42)**		**15-year- olds (*n* = 24)**		**University students (*n* = 53)**		**Parents (*n* = 66)**	
	r	rho	r	rho	r	rho	r	rho
**proximal segments:**								
MPI/mMPI (proximal)	**(0.50)**	0.54	**0.48**	0.57	**(0.52)**	0.51	**(0.30)**	0.32
sMPI (proximal)	**(0.47)**	0.53	**(0.41)**	0.49	**(0.45)**	0.44	**(0.25)**	0.31
**cervical segments:**								
MPI (cervical)	**0.48**	0.47	**0.63**	0.59	**0.47**	0.48	**0.49**	0.34
mMPI/sMPI (cervical)	**0.42**	0.39	**0.61**	0.56	**0.44**	0.44	**0.41**	0.29

Brackets indicate that the assumption of normal distribution is violated and that the respective correlations are not compared with those of the MPI with the PBI

### Convergent validity

Correlation data for the plaque indices are shown in Table 3 for the 10 and 15-year-olds, and in Table 4 for the students and parents. All correlations are statistically significant (all *p* < 0.001).

In all groups, the scales that refer to plaque at the gingival margin (i.e. the variants of the MPI and the PCR), show very high inter-correlations of r ≥ 0.94. The correlation with the TQHI (that also scores plaque distant of the gingival margin) tends to be slightly lower (0.80 ≤ r ≤ 0.94).

### Concurrent validity

Table 5 shows the correlations of the indices with the PBI. The TQHI and the MPI correlate equally with the PBI in all groups (all *p* > 0.05). The lowest correlations with the PBI are found among parents, where there are also larger differences between the different indices, with the simplified forms of the MPI and the PCR having lower correlations with the PBI than the MPI (all *p* < 0.001).

Table 6 shows the correlations between the PBI and the subscales of the MPI measures considering only proximal and only cervical segments of the gingival margin. The correlations of the cervical segments with the PBI are not different from those of the MPI with the PBI (all *p* > 0.05). For the proximal segments, the assumption of normal distribution isviolated in most cases, so the correlations cannot be compared by inferential statistics. The correlation of the MPI (proximal) of the 15-year-olds with the MPI is not significantly different from that of the MPI with the PBI (*p* = 0.0618).

## Discussion

This research assessed whether simplified versions of the MPI [5] would yield comparable results to the original version. It also assessed the convergence of the indices derived from the MPI (MPI, mMPI, sMPI) with the PCR [4] and the TQHI [2], and compared the concurrent validity of the MPI in relation to the PBI [9, 14] with that of the mMPI, sMPI, PCR, and TQHI. Data from 4 groups with different age and demographic characteristics were analyzed. In all groups, plaque was assessed immediately after oral hygiene, which the participants were asked to perform to the best of their ability.

All plaque indices analyzed here show a high degree of convergence. The convergence within the indices limited to the gingival margin (MPI, mMPI, sMPI, PCR) is close to r = 1.0. This means that they are interchangeable for assessing plaque as a result of oral hygiene. Slightly lower correlations of these indices with the index that also assesses plaque in the more coronal parts of the crown (TQHI; [2]) indicate that the marginal and total crown indices are not equally interchangeable. Nevertheless, there is a high degree of convergence. It may therefore be appropriate to apply criteria such as practicality when deciding which of these indices should be used to measure plaque by hygiene. The TQHI is more difficult to train, as it requires the examiner to assess not only the presence or absence of plaque but also its expansion. In addition, the description in the original literature leaves room for interpretation. On the one hand, it states that a score of 3, 4 and 5 should be given if one, two and three-thirds of the crown are covered with plaque respectively [2]. This could be interpreted to mean the entire surface of the crown. On the other hand, the authors refer to the original work of Quigley and Hein [3], where the expansion describes how far the plaque extends from the gingival margin into the crown. This would mean that a score of 4 would be given if the plaque reached the second third of the crown, even if in total only one third of the crown is covered by plaque. Such ambiguities not only make it difficult to calibrate the examiners. They also reduce the comparability of the results between different working groups. The four indices that assess the presence or absence of plaque in pre-specified segments of the gingival margin leave less room for interpretation. However, they require more effort, as they record significantly more data. The present analysis shows, that the amount of data can be reduced without loss of convergent validity. The very high correlations between PCR and sMPI also show that the disadvantage of the PCR, namely the need to detect plaque on the inner and outer surfaces of a tooth simultaneously, can be overcome.

These considerations relate to research situations where it is only important to record the residual plaque after oral hygiene. However, plaque is often measured to determine the risk of plaque-related diseases such as gingivitis. Therefore, the concurrent validity of the different plaque indices with a common measure of gingivitis, the PBI, was also assessed. Plaque and gingivitis measure different things. Plaque assesses the immediate effects of oral hygiene measures. Gingivitis only emerges when plaque persists for a longer time and plaque changes its characteristics [16]. Therefore, perfect correlations are not expected. However, it is expected that the correlations will be in the medium range [5, 17]. The results largely confirm this assumption, although in the parent group the simplified versions of the MPI and the PCR appear to perform worse in this respect than the original MPI. There is no easy explanation of this. However, the correlations of the MPI and the TQHI with the PBI are also lower in this group than in the others. Among the groups analyzed here, the group of parents has the widest age range and the highest average age. It is possible that the influence of factors other than plaque on the PBI increases with age. This could lead to lower correlations and make the concurrent validity of the assessment more sensitive to a reduction in the amount of data assessed. Another study also showed that the correlations of plaque with bleeding on probing (BOP) are lower in periodontitis patients than in other groups [17]. Still, more research is needed in this regard.

The current study also attempted to assess whether the subscales of the MPI and its simplified forms that relate to plaque on the distal and mesial (proximal) segments of teeth versus the cervical segments would differ in their correlation with the PBI from the total MPI. No significant differences were found for the cervical subscales. For the proximal subscales such analyses were not possible for statistical reasons.

The strengths of the current study are the highly standardized conditions under which the data were assessed, the fact that the data refer to different studies with different examiners, and the fact that all examiners were thoroughly calibrated and had to meet a strict calibration criterion before they were allowed to assess the data. However, there are several limitations that should be taken into account when interpreting the data. First, none of the groups studied was a representative sample of the population. This limits the generalizability of the results. Second, simplified versions of the MPI and PCR were derived from the MPI assessments and were not recorded independently. However, it would be difficult to record these independently in vivo, as it would require a number of different examiners to assess the same situation. Yet the situation could easily change between two examiners, as plaque staining can fade. Another approach would be to look at intra-oral scanning data of plaque distribution, rather than relying on in vivo examinations. One way to do so would be not to rely on the immediate results of a clinical examination but to examine intra-oral scanning data of plaque distribution. However, this assumes that it would be possible to make a reliable assessment of the plaque at the gingival margin. To date, this appears to be difficult [18, 19], although further technical refinement of scanning methods may allow such studies in the near future.

In conclusion, the mMPI and sMPI provide a reliable, time-efficient alternative to more complex plaque scoring methods. Their ease of use could make them particularly valuable in both clinical settings and large-scale epidemiological studies where time constraints are a significant factor.

## Conclusion

The current study shows that the simplified versions of the MPI have high convergent validity with its long form and other plaque indices. Their concurrent validity with the PBI is similar to that of the MPI in younger age groups. In a more heterogeneous middle-aged group, the long form has better concurrent validity than the simplified forms.

## Data Availability

The datasets generated and/or analysed during the current study are not publicly available due to privacy reasons but are available from the corresponding author on reasonable request.

## Abbreviations

DMFT/dmft: Decayed, missing filled teeth
MPI: Marginal Plaque Index
mMPI: Modified Marginal Plaque Index
sMPI: Short Marginal Plaque index
PBI: Papillary Bleeding Index
PCR: Plaque Control Record
RMNPI: Refinement of the Modified Navy Plaque Index
TQHI: Turesky Modification of the Quigley & Hein Index
